# Supplementary material for: Utilization of Monosaccharides by Hungateiclostridium thermocellum ATCC 27405 through Adaptive Evolution
Source: Microorganisms. 2021 Jul 4;9(7):1445. doi: 10.3390/microorganisms9071445 (PMC8303734; doi:10.3390/microorganisms9071445)
Supplement: Supplementary file 1 [file microorganisms-09-01445-s001.zip › microorganisms-1281237 - Supplementary Materials.pdf]

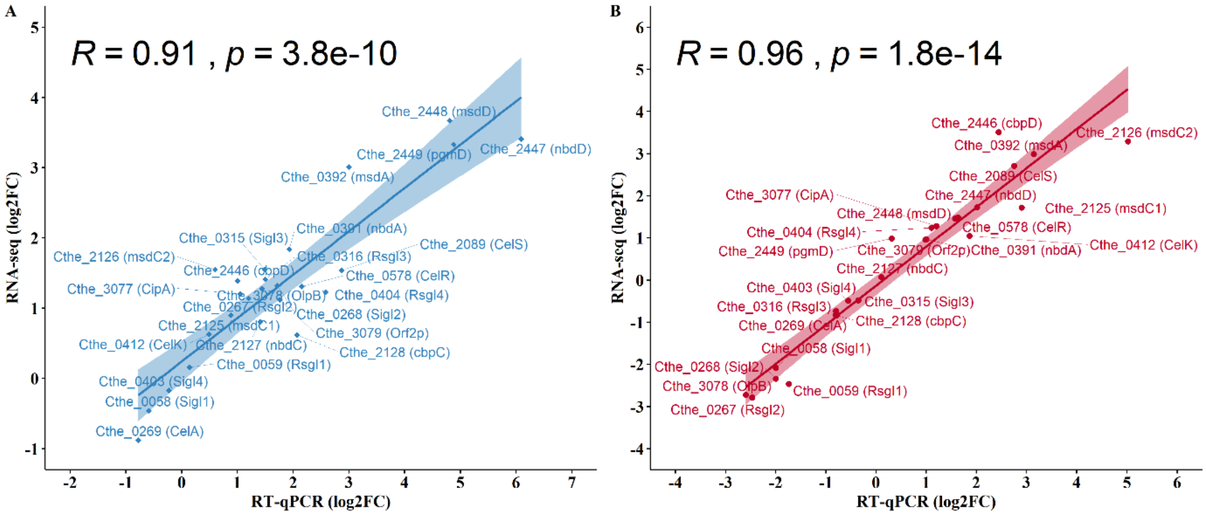

Figure S1 Correlation between RNA-seq and RT-qPCR results in FAs1, GAs1, and CGs1. **(A)** Comparison of gene expression measurements of FAs1 relative to CGs1. **(B)** Comparison of gene expression measurements of GAs1 relative to CGs1. Twenty five genes including *nbdA* (Cthe_0391), *msdA* (Cthe_0392), *CipA* (Cthe_3077), *OlpB* (Cthe_3078), *Orf2p* (Cthe_3079), *CelS* (Cthe_2089), *CelK* (Cthe_0412), *CelR* (Cthe_0578), *CelA* (Cthe_0269), *SigI1* (Cthe_0058), *RsgI1* (Cthe_0059), *SigI2* (Cthe_0268), *RsgI2* (Cthe_0267), *SigI3* (Cthe_0315), *RsgI3* (Cthe_0316), *SigI4* (Cthe_0403), *RsgI4* (Cthe_0404), *cbpD* (Cthe_2446), *nbdD* (Cthe_2447), *msdD* (Cthe_2448), *pgmD* (Cthe_2449), *msdC1* (Cthe_2125), *msdC2* (Cthe_2126), *nbdC* (Cthe_2127), *cbpC* (Cthe_2128) were chosen to determine their levels of expression compared with their values in RNA-seq data.

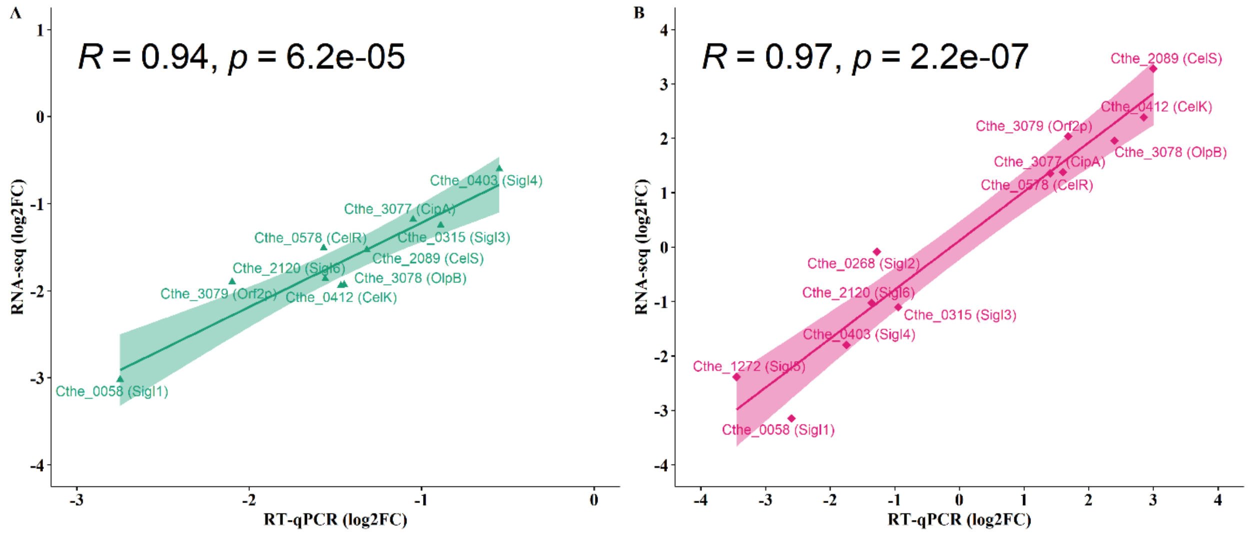

Figure S2 Correlation between RNA-seq and RT-qPCR results in FAs8, GAs8, and CGs1. **(A)** Comparison of gene expression measurements of FAs8 relative to CGs1. **(B)** Comparison of gene expression measurements of GAs8 relative to CGs1. Twelve selected genes including Cthe_0058 (*SigI1*), Cthe_0268 (*SigI2*), Cthe_0315 (*SigI3*), Cthe_0403 (*SigI4*), Cthe_1272 (*SigI5*), Cthe_2120 (*SigI6*), Cthe_0412 (*CelK*), Cthe_0578 (*CelR*), Cthe_2089 (*CelS*), Cthe_3077 (*CipA*), Cthe_3078 (*OlpB*), Cthe_3079 (*Orf2p*) were determined the levels of expression compared with their values in RNA-seq data.
